# Preeclampsia and neonatal outcomes in adolescent and adult patients

**DOI:** 10.25122/jml-2022-0264

**Published:** 2022-12

**Authors:** Ana Veronica Uzunov, Diana Cristina Secara, Claudia Mehedințu, Monica Mihaela Cîrstoiu

**Affiliations:** 1 Department of Obstetrics and Gynaecology, Carol Davila University of Medicine and Pharmacy, Bucharest, Romania; 2 Department of Obstetrics and Gynaecology, University Emergency Hospital Bucharest, Bucharest, Romania; 3 Department of Obstetrics and Gynaecology, Clinical Hospital of Obstetrics and Gynaecology Filantropia, Bucharest, Romania

**Keywords:** preeclampsia, adolescents, neonatal outcome, birth weight, preterm

## Abstract

Preeclampsia is an important health problem with a higher prevalence in the adolescent population. Furthermore, preeclampsia causes adverse maternal and neonatal outcomes. Newborns can be affected by preeclampsia, resulting in lower birth weight or Apgar score, the need for neonatal intensive care, or prematurity. All these complications are also associated with adolescent pregnancies, and together with preeclampsia, it can determine poorer neonatal outcomes. The aim of the study was to compare the neonatal outcomes of adolescents and adults with preeclampsia. We analyzed data on all the newborns of adolescents with preeclampsia (n=12) who delivered at the Department of Obstetrics and Gynecology of University Emergency Hospital in Bucharest between January 1^st^, 2019, and December 31^st^, 2019 and compared it with data from 12 aleatory newborns of adults diagnosed with preeclampsia. The prevalence of preeclampsia was higher in the adolescent population compared with the adult one. The weight of newborns was lower among adolescents with preeclampsia. There were no significant differences in Apgar scores between the two groups. Preterm delivery was more frequent in adolescent patients with preeclampsia. Preeclampsia is an additional risk factor for adolescent pregnancy, but it is also a severe materno-fetal complication for this population.

## INTRODUCTION

Adolescence is defined as the period of life between 10 and 19 years [1, 2]. Most pregnancies that occur during this period are unintended [1, 2]. Worldwide, approximately 15% of women under 18 years old gave birth between 2015–2020. Furthermore, more than 90% of these deliveries occurred in low- and middle-income countries [1–3]. World Health Organization (WHO) estimates that globally, approximately 21 million adolescents aged between 15 and 19 years get pregnant yearly, of whom 12 million end up giving birth [1, 4].

Adolescent pregnancy has a higher risk of obstetrical and neonatal complications than adults. Therefore, regarding the obstetrical risks, adolescents have an increased risk of preeclampsia, premature rupture of the membranes, anemia, sexually transmitted diseases, and maternal deaths [1, 5–7]. In what concerns neonatal outcomes, newborns of adolescent patients have a higher risk of prematurity, stillbirths, low birth weight, lower Apgar score, and congenital anomalies [1, 2, 6, 8, 9].

Preeclampsia is a progressive hypertensive disorder during pregnancy that can have an important impact involving multiple organs. It used to be defined as the presence of hypertension, blood pressure ≥140 mmHg systolic and ≥90 mmHg diastolic, diagnosed for the first time after 20 weeks of gestation, plus proteinuria and edema [10]. Over the years, the physiopathology of preeclampsia has become better understood; therefore, the definition includes hypertension and organ dysfunction in the kidneys, liver, neurologic, hematological, or uteroplacental systems [11]. Risk factors for preeclampsia are primiparity, pregnancy at an early or advanced age, and a history of preeclampsia or associated comorbidities [10, 12].

Multiple studies have explored the relationship between preeclampsia and the adolescent population, and most of them concluded that this pathology is more frequent in teenagers than in older patients [1, 12–14]. This pathology exposes newborns to adverse outcomes such as prematurity, low birth weight, and fetal distress [12, 15, 16].

Some of the causes for the high rates of preeclampsia among adolescents may include uterine immaturity leading to defective deep placentation or the increased prevalence of obesity among teenagers [17].

This study aimed to investigate and compare the neonatal outcomes of adolescents and adults with preeclampsia.

## MATERIAL AND METHODS

We performed an observational, retrospective study that included 12 adolescent patients diagnosed with preeclampsia who delivered at the Department of Obstetrics and Gynecology of the University Emergency Hospital in Bucharest between January 1^st^, 2019, and December 31^st^, 2019, and a control group of 12 patients aged between 20 and 24 years diagnosed with preeclampsia who delivered in the same period in the same unit. The control group was selected randomly from patients diagnosed with preeclampsia and without other pathological conditions. The diagnosis of preeclampsia was established according to actual protocols. The data regarding neonatal outcomes were collected from hospitalization sheets and the Base Data System of the University Emergency Hospital in Bucharest.

Inclusion criteria in the study were: patients aged 14–24 years old who delivered at the University Emergency Hospital in Bucharest and who were diagnosed with preeclampsia. Exclusion criteria were the refusal to sign the consent or the presence of other pathologies that could influence neonatal outcomes.

The mother's age was recorded at the time of delivery. The gestational age was ascertained by the last menstruation period. Delivery was defined after the fetus completed 24 weeks of gestation or weighed over 500 grams. The neonatal status was established by the neonatal team and included fetal weight and 1-minute Apgar score, the need for neonatal intensive care, preterm births, or stillbirths.

## RESULTS

**Figure 1:**
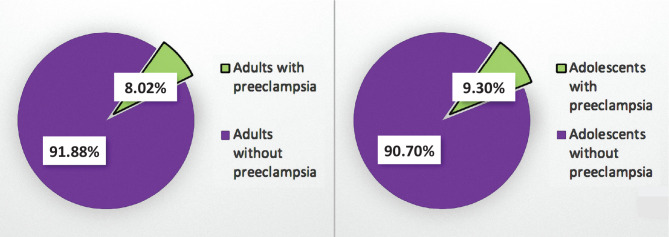
The rate of preeclampsia among adolescents and adults who delivered at the Department of Obstetrics and Gynecology of the University Emergency Hospital in Bucharest between January 1^st^, 2019, and December 31^st^, 2019.

129 adolescent patients gave birth at the Department of Obstetrics and Gynecology of the University Emergency Hospital in Bucharest between January 1^st^, 2019, and December 31^st^, 2019, representing 5.30% of all deliveries in our unit during that period. Out of these, 12 adolescents (9.30%) were diagnosed with preeclampsia. Among adult patients who delivered in our unit (n=2306), 8.02% had preeclampsia (Figure 1).

**Figure 2:**
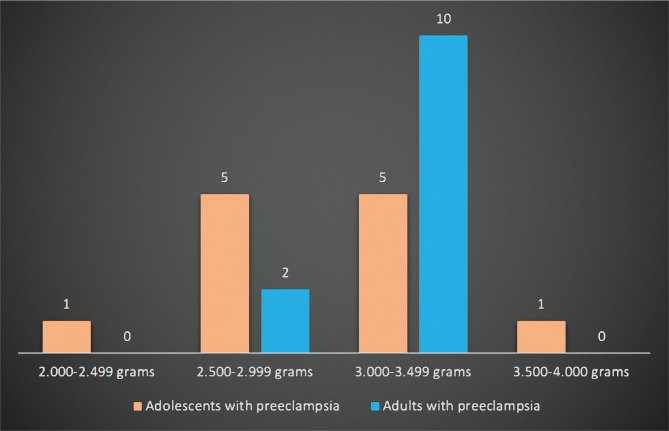
The birth weight of newborns of adolescents with preeclampsia compared to adults with preeclampsia.

Regarding neonatal outcomes, we first analyzed and compared the birth weight of newborns of adolescent and adult patients with preeclampsia (Figure 2). There were no newborns under 2000 grams nor over 4000 grams in any of the groups. Six newborns (50.00%) weighed under 3000 grams in the study group, while in the control group, there were only two newborns (16.67%) under this weight. Most newborns in the study group (83.33%) weighed between 3000 and 3499 grams.

**Figure 3:**
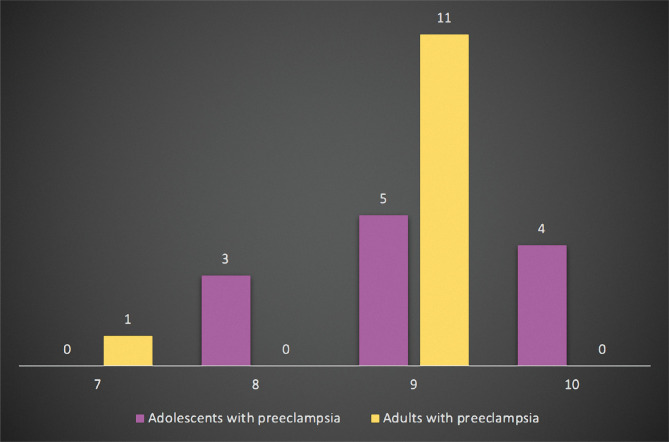
The one-minute Apgar score of newborns of adolescents with preeclampsia compared to adults.

Furthermore, we evaluated the Apgar score calculated one minute after birth (Figure 3). There were no newborns with Apgar scores under 7 in any of the groups. In the study group, there were three newborns (25.00%) with an Apgar score of 8 and four with an Apgar score of 10 (33.33%). Most of the newborns from adolescents with preeclampsia had an Apgar score of 9 (41.67%). In the control group, there was one newborn (8.33%) with Apgar 7, none with Apgar 8 or 10, and the majority of newborns of adults with preeclampsia (91.67%) had an Apgar score of 9.

None of the participants in the two groups had stillbirths or newborns with congenital malformations. Also, none of the newborns needed neonatal intensive care.

Regarding prematurity, in the study group, two newborns (16.67%) were born before 37 weeks of gestation, while in the control group, there was no case.

**Figure 4:**
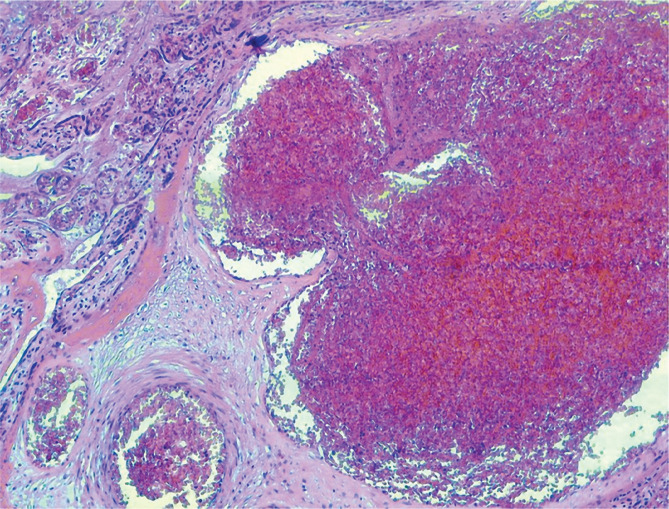
Placenta from an adolescent with preeclampsia – fibrin deposition in the large blood vessels with incipient thrombus.

The histopathological appearances of the placenta from an adolescent patient with preeclampsia are presented in Figure 4.

## DISCUSSION

The prevalence of preeclampsia is estimated to be 5%, but the rate varies according to multiple factors such as age, comorbidities, parity, or ethnic features [18–20]. A systemic review and meta-analysis, which analyzed over 290000 adolescents with preeclampsia and eclampsia, showed that the prevalence of preeclampsia in this population was 5.9% [10]. Another study that evaluated 411 teenagers found that preeclampsia complicated 16.78% of all cases [21]. 9.30% of all adolescents who delivered in our unit between January 1^st^, 2019, and December 31^st^, 2019, had preeclampsia. Compared to adult patients, whose rate was 8.02%, adolescents had a higher prevalence of preeclampsia.

Regarding the newborn's outcomes, studies show that women with preeclampsia have newborns with lower birth weights [22, 23]. In our study, 50% of adolescents gave birth to a newborn under 3000 grams compared to the control group, where only 16.67% of the newborns weighed under 3000 grams. Studies show that adolescents tend to deliver newborns with lower birth weights than adults [2, 14, 24]. Also, a lower Apgar score is associated with women with preeclampsia [22, 23]. In our study, in both groups, most of the newborns from patients with preeclampsia had an Apgar score of 9 and 10.

Nevertheless, 25% of newborns had an Apgar score under 9 in the study group, while in the control group, the percentage was 8.33%. So, the newborns of adolescents with preeclampsia had a lower 1-minute Apgar score compared to those of adult patients with preeclampsia. None of the newborns from both groups were admitted to the neonatal intensive care unit. There were no stillbirths in any of the groups.

Preterm birth represents a well-known neonatal complication associated with preeclampsia [22, 23]. In our study, there were no cases of prematurity in the control group, while in the study group, there were two cases (16.67%). However, preterm birth is also associated with adolescent pregnancy [2, 14, 24]. The causes for the high incidence of preterm delivery among adolescents are still unclear, but some mechanisms are supposed to be involved. Of these, the most discussed ones are the immaturity of the uterine and cervical blood supply [14].

## CONCLUSION

Preeclampsia is an important health problem affecting adolescents and adults. The neonatal outcome, representing the 1-minute Apgar score and the newborns' weight, depends on the maternal age, but it also may be influenced by preeclampsia. Therefore, preeclampsia represents an important additional factor for neonatal status in the adolescent population.

The study has some limitations, represented by the small size of samples and the lack of pregnancy information among adolescent patients due to poor antenatal care.

## Data Availability

Further data is available from the corresponding author on reasonable request.
